# Mitral valve repair in severe mitral regurgitation due to Barlow’s disease with concomitant mitral annular disjunction: a case report

**DOI:** 10.1186/s44215-025-00196-4

**Published:** 2025-03-04

**Authors:** Koji Furukawa, Ayaka Iwasaki, Hirohito Ishii, Sakaguchi Shuhei, Kousuke Mori, Shohei Hiromatsu

**Affiliations:** Division of Cardiovascular Surgery, Department of Surgery, Faculty of Medicine, University of Miyazaki, 5200 Kiyotakecho Kihara, Miyazaki City, Miyazaki 889-1692 Japan

**Keywords:** Mitral annular disjunction, Mitral regurgitation, Barlow’s disease, Mitral valve repair

## Abstract

**Background:**

Mitral annular disjunction (MAD) is characterized by the detachment of the mitral valve-left atrial junction from the left ventricular myocardium. The association of MAD with Barlow’s disease and its relevance to treatment are increasingly recognized.

**Case presentation:**

A 75-year-old male with a history of mitral regurgitation (MR) and ablation for paroxysmal atrial fibrillation was diagnosed with severe MR due to Barlow’s disease, as confirmed by echocardiography. Imaging revealed disjunction at the mitral valve’s posterior annulus. During surgery, the posterior leaflet was resected along the annulus with precise height adjustments. A 6-mm separation between the mitral valve–left atrial junction and the left ventricular myocardium was sutured using a four-stitch mattress technique. The procedure included leaflet repair, insertion of artificial chordae, and mitral annuloplasty. Postoperatively, the MAD was corrected successfully, eliminating the severe MR.

**Conclusions:**

Confirming the presence of MAD before surgery is essential for patients with MR. Surgical correction of MAD is imperative when present to address the disjunction effectively.

**Supplementary Information:**

The online version contains supplementary material available at 10.1186/s44215-025-00196-4.

## Background

Mitral annular disjunction (MAD) is a structural cardiac anomaly characterized by detachment of the mitral valve (MV)–left atrial junction from the left ventricular myocardium [1–8]. Its association with Barlow’s disease (BD) has attracted significant interest [4–8]. However, there are no standard guidelines for determining the need for surgery in different cases of MAD and for managing MAD. This report discusses a case in which MV repair, including MAD closure, effectively addressed mitral regurgitation (MR) caused by BD. Additionally, we provide an in-depth analysis of the BD–MAD association, highlighting its pathological significance, implications for surgical intervention, and prospective challenges.

## Case presentation

A 75-year-old male with a history of MR diagnosed over 20 years ago and prior catheter ablation for paroxysmal atrial fibrillation (performed 5 years ago) presented to our department for management of worsening MR.

**Fig. 1:**
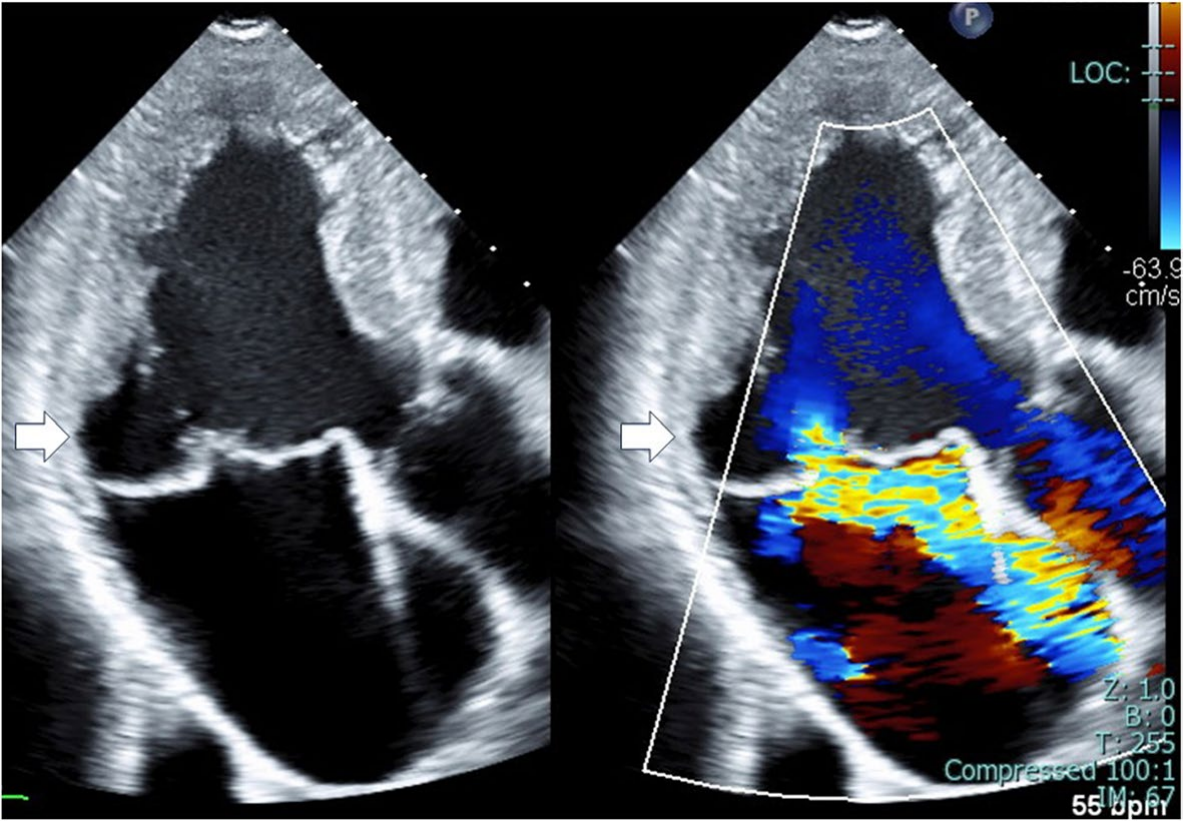
Transthoracic echocardiograms show an enlarged mitral annulus, mitral valve billowing with posterior leaflet prolapse and mitral annular disjunction (white arrows), and severe mitral regurgitation

Upon admission, his height (176 cm), weight (62 kg), blood pressure (123/91 mmHg), and heart rate (64 beats/min) were recorded. Auscultation revealed a grade 4/6 systolic murmur audible at the cardiac apex. Blood chemistry showed a brain natriuretic peptide level of 163 pg/mL. Chest radiography indicated a cardiothoracic ratio of 50%. Electrocardiography showed sinus rhythm with premature ventricular contractions and a first-degree sinoatrial block. Transthoracic echocardiography revealed severe MR with anterior deviation due to mitral annular enlargement and MV billowing with posterior leaflet prolapse. Additionally, the echocardiography indicated MAD at the posterior MV (Fig. 1 and Additional file 1).

The left ventricle’s systolic and diastolic diameters were 64 and 45 mm, respectively, with a left ventricular ejection fraction of 54%. The left atrium diameter was 54 mm. Mild tricuspid regurgitation was observed, with a tricuspid annular diameter of 38 mm (21.5 mm/m^2^) in the apical four-chamber view. Transesophageal echocardiography showed MV billowing with posterior leaflet prolapse, MR, and MAD. Cardiac catheterization revealed a mean pulmonary capillary wedge pressure of 12 mmHg and mean pulmonary artery pressure of 16 mmHg. Coronary angiography indicated that the coronary arteries had no significant stenosis.

The patient was diagnosed with severe MR, consistent with BD and associated with MAD. Surgery was performed to treat the MR, which was progressively enlarging the heart and compromising cardiac function.

**Fig. 2:**
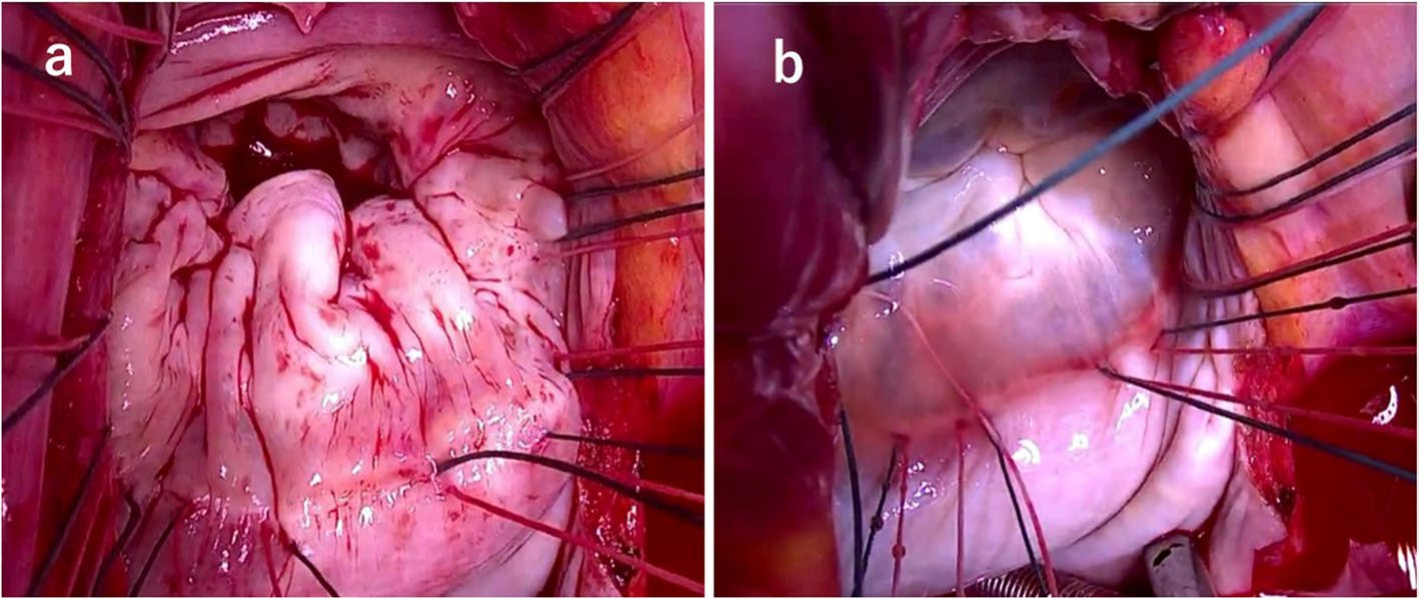
Intraoperative findings. **a** An elongated posterior leaflet and a dilated mitral annulus. **b** A water test shows effective valve coaptation; the posterior leaflet extends over a significant area of the valve orifice

Under general anesthesia, a cardiopulmonary bypass (CPB) was initiated by inserting cannulas into the ascending aorta, superior vena cava, and inferior vena cava. An aortic cross-clamp was applied, and antegrade blood cardioplegia was used to induce cardiac arrest. Given the patient’s history of paroxysmal atrial fibrillation, the left atrial appendage was resected using an automatic stapler, with no thrombus detected. Access to the MV was achieved through an incision on the left atrium’s right side. The MV showed significant annular dilation, and the P2 posterior leaflet was notably redundant, measuring 28 mm in height (Fig. 2a). No chordal rupture was observed; however, the P2 marginal chordae were slightly elongated. Despite the posterior leaflet enlargement, the other leaflets and chordae tendineae showed no organic changes or abnormalities.

**Fig. 3:**
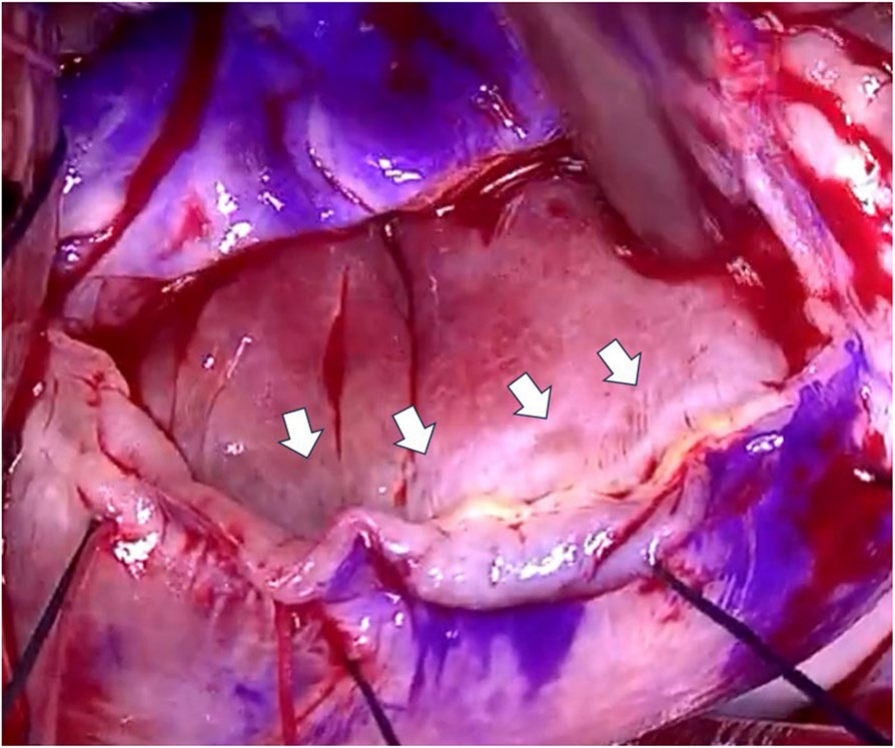
Intraoperative findings show separation between the left ventricular wall and the mitral valve-left atrium junction (white arrows)

**Fig. 4:**
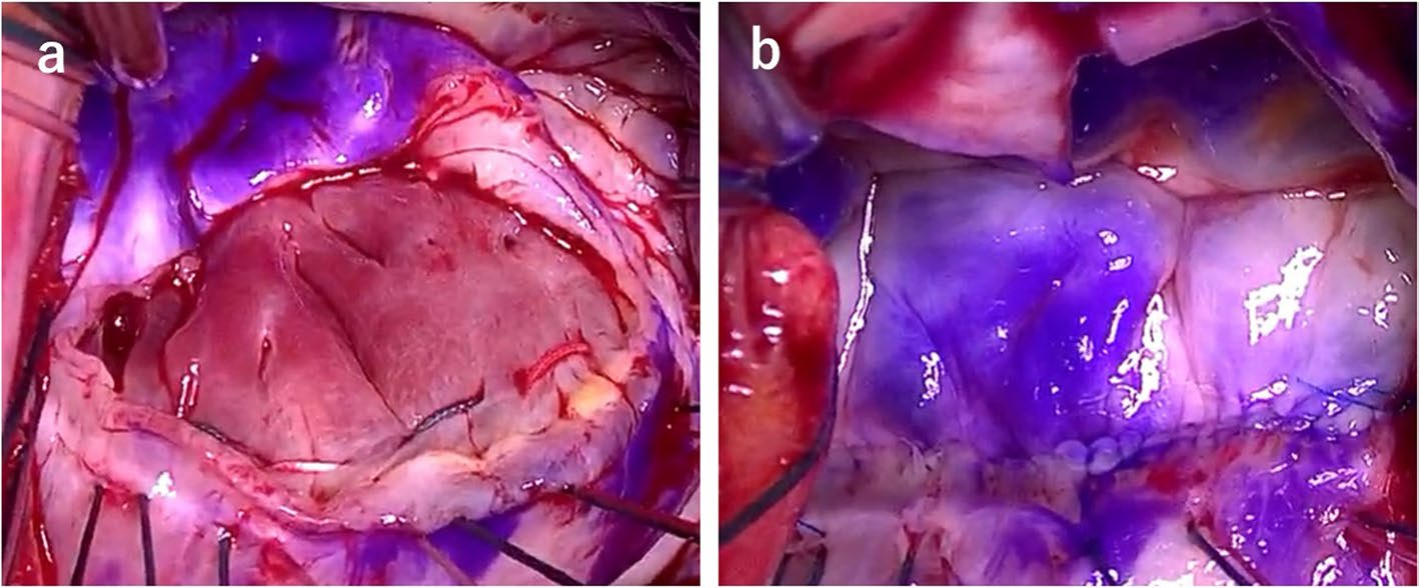
Intraoperative findings. **a** Sutures are placed through the left ventricular wall to repair the mitral annular disjunction. **b** After resuturing, the posterior leaflet is correctly adjusted in height

Annuloplasty sutures were placed from the left to right fibrous trigones across the posterior annulus, and regurgitation was assessed. Although MR was controlled, the posterior leaflet remained larger than the anterior leaflet (Fig. 2b). Consequently, a 3-cm incision was made in the posterior leaflet’s P2 segment near the annulus, followed by a 4-mm wedge resection. Examination of the space beneath the posterior annulus revealed a separation between the left ventricular wall and the MV–left atrium junction (Fig. 3). To seal the separation, we placed four sets of non-everting mattress sutures using 4–0 polyester thread from the left ventricular wall to the left atrial wall at the leaflet junction (Fig. 4a). The trimmed leaflets were sutured using a continuous 5–0 monofilament, reducing the posterior leaflet height to approximately 18 mm (Fig. 4b). Water testing demonstrated an improved anterior-to-posterior leaflet ratio of approximately 2:1.

A pair of artificial chordae tendineae were implanted at the P2 position to improve valve coaptation. A 38-mm SimuPlus band (Medtronic Inc., Minneapolis, MN, USA) was chosen to correspond with the MV aperture, followed by a tricuspid annuloplasty using a 30 mm Tri-Ad band (Medtronic Inc., Minneapolis, MN, USA). Transesophageal echocardiography confirmed the resolution of MAD, and the MR was effectively managed. The patient was weaned off CPB without complications. The aortic cross-clamping, total CPB, and entire surgery durations were 188, 230, and 409 min, respectively.

The patient’s postoperative recovery was uneventful, and he was discharged on the 20th day after surgery. At the 6-month follow-up, electrocardiography showed sinus rhythm without premature ventricular contractions. Transthoracic echocardiography revealed a left ventricular ejection fraction of 51%. The left ventricular diastolic and systolic diameters had decreased to 55 mm and 40 mm, respectively, and the left atrial diameter had reduced to 47 mm. No MAD or MV leaflet billowing was detected, and the MR was successfully controlled (Additional file 2). The patient remains asymptomatic and under continued observation.

## Discussion and conclusions

BD is a clinical syndrome often identified by a non-ejective click due to late systolic MR and a corresponding murmur [9, 10]. BD involves significant annular dilation, excessive valvular tissue, leaflet billowing, and myxomatous degeneration [9–11]. MV repair in these cases is generally more complex than in cases of fibroelastic deficiency [9]. Surgical outcomes may be improved by a better understanding of the MR mechanisms in BD, informed by clinical experience and advancements in diagnostic imaging.

The development of BD is associated with abnormal mitral annulus movements, including unusual widening and flattening during the late systolic phase and compensatory enlargement of MV leaflets [3–7, 10–12]. These leaflets protrude into the left atrium and produce a systolic click. Inadequate compensation may cause functional MV prolapse, which is indicated by a central regurgitation jet. In contrast, excessive stress can cause chordal elongation or rupture and organic MV prolapse, as identified by an eccentric regurgitation jet. MAD is also a significant factor in abnormal mitral annulus movement.

The MV’s posterior leaflet is typically anchored to the mitral annulus, which demarcates the left ventricle and atrial walls. This anchorage enables the MV annulus to contract synchronously with the movements of the left ventricular muscles, reducing its systolic size, forming a saddle shape, and ensuring complete coaptation of the leaflets [3, 4]. In patients with MAD, the mitral annulus fails to follow the left ventricular wall contractions; instead, it abnormally dilates and flattens during systole. This dysfunction contributes to the deterioration and functional alterations of the MV complex associated with BD [3–5, 7, 10, 12]. The high incidence of MAD among patients with MV prolapse, especially in patients with BD, corroborates this hypothesis [6–8]. Additionally, dysfunction of the MV complex due to MAD can cause fibrosis of the left ventricular papillary muscles, leading to lethal ventricular arrhythmias [6],

In MR due to BD, MV repair involves managing abnormal annular movements with annuloplasty, preventing the systolic anterior motion of the MV, and addressing structural deterioration of the MV leaflets and chordae tendineae through resection or chordal reconstruction [9–13]. When MAD is present, ensuring a reliable reduction of MAD is crucial [1–4, 7, 8]. Essayagh et al. reported that although the presence of MAD itself does not affect the repairability of prolapsed MV, patients with residual MAD after conventional MAP had a significantly longer preoperative MAD distance than those without (9 ± 5 vs. 3 ± 4 mm, *p* < 0.0001) [7]. In our case, the posterior leaflet was resected along the annulus, and the leaflet height was meticulously controlled. Subsequently, annuloplasty sutures were placed through the resected area to close the MAD, and an annuloplasty ring was applied. These modified annuloplasty techniques, which directly address the MAD, have shown promising early results [1, 2]. When choosing an annuloplasty ring, the principle is to avoid using small rings to prevent the systolic anterior motion of the MV [9, 10, 13]. The difference in surgical efficacy between ring types remains unclear [14], and some surgeons prefer semi-rigid types in the hope of a remodeling effect [12]. However, we used a flexible band in accordance with previous reports [2, 11].

However, the long-term outcomes of MV repair in BD with MAD may be less favorable compared to those in cases of posterior leaflet prolapse due to fibroelastic deficiency, likely due to more advanced degenerative changes [2, 8]. In contrast, for patients with MR and MAD with ventricular arrhythmias, early surgery may reduce the number of arrhythmic events and improve prognosis [6]. However, there are no standard guidelines for determining the need for surgery in different cases of MAD or for managing MAD. This uncertainty also applies to the choice of MV intervention, such as minimally invasive surgery, including small incisions, thoracoscopic and robotic MV surgery, or transcatheter MV repair in patients with MAD, highlighting the need for further research.

In summary, MAD significantly affects the progression of BD. Determining the presence of MAD using preoperative imaging and securing closure during surgical procedures is imperative.

## Supplementary Information

Additional file 1. Movie of preoperative transthoracic echocardiograms showing mitral annular disjunction and severe mitral regurgitation.

Additional file 2. Movie of follow-up transthoracic echocardiograms showing no mitral annular disjunction and good control of mitral regurgitation.

## Data Availability

Not applicable.

## Abbreviations

MAD: Mitral annular disjunction
MR: Mitral regurgitation
MV: Mitral valve
BD: Barlow’s disease
CPB: Cardiopulmonary bypass
